# Sterol-activated amyloid beta fibril formation

**DOI:** 10.1016/j.jbc.2023.105445

**Published:** 2023-11-08

**Authors:** Ian Cook, Thomas S. Leyh

**Affiliations:** Department of Microbiology and Immunology, Albert Einstein College of Medicine, Bronx, New York, USA

**Keywords:** Alzheimer’s disease, cholesterol sulfate, cholesterol, amyloid beta plaque, structure, monomer, dimer, fibril, molecular dynamics, mechanism, Aβ_42_ peptide, sulfotransferase 2B1b

## Abstract

The metabolic processes that link Alzheimer’s disease (AD) to elevated cholesterol levels in the brain are not fully defined. Amyloid beta (Aβ) plaque accumulation is believed to begin decades prior to symptoms and to contribute significantly to the disease. Cholesterol and its metabolites accelerate plaque formation through as-yet-undefined mechanisms. Here, the mechanism of cholesterol (CH) and cholesterol 3-sulfate (CS) induced acceleration of Aβ_42_ fibril formation is examined in quantitative ligand binding, Aβ_42_ fibril polymerization, and molecular dynamics studies. Equilibrium and pre-steady-state binding studies reveal that monomeric Aβ_42_•ligand complexes form and dissociate rapidly relative to oligomerization, that the ligand/peptide stoichiometry is 1-to-1, and that the peptide is likely saturated *in vivo*. Analysis of Aβ_42_ polymerization progress curves demonstrates that ligands accelerate polymer synthesis by catalyzing the conversion of peptide monomers into dimers that nucleate the polymerization reaction. Nucleation is accelerated ∼49-fold by CH, and ∼13,000-fold by CS — a minor CH metabolite. Polymerization kinetic models predict that at presumed disease-relevant CS and CH concentrations, approximately half of the polymerization nuclei will contain CS, small oligomers of neurotoxic dimensions (∼12-mers) will contain substantial CS, and fibril-formation lag times will decrease 13-fold relative to unliganded Aβ_42_. Molecular dynamics models, which quantitatively predict all experimental findings, indicate that the acceleration mechanism is rooted in ligand-induced stabilization of the peptide in non-helical conformations that readily form polymerization nuclei.

Clinical studies estimate that 12% of United States citizens aged 65 and older suffer from Alzheimer’s dementia (1) and 34% of those over age 85 have the disease. Collateral consequences of the disease include approximately 15 billion hours of annual caregiving by family and non-family members—a considerable societal stress. The fiscal burden of Alzheimer’s disease (AD) in 2022 is estimated at 0.6 trillion dollars in the United States and in the absence of mitigating factors is expected to rise as the majority of the baby-boom generation enters the over-65 age category.

Roughly 15 years (2, 3) prior to the onset of symptoms, AD is thought to begin with the dysregulation of metabolic processes that include elevated production of Aβ peptides—a quintet of overlapping peptides (36–42-mers) proteolytically clipped by β- and γ-secretase from a trans-membrane segment of the amyloid precursor protein (APP) (4). Aβ peptides are amyloidogenic — they self-organize into dimers (polymerization nuclei) that rapidly add monomers to form oligomers, proto-fibrils, fibrils, and ultimately dense nests of fibrils known as senile plaques. The preponderance of evidence over the last several decades (5, 6, 7) supports that certain, as yet undefined small soluble Aβ oligomers, 2- to 12-mers (8) are the primary neurotoxic forms of the peptide. Cytotoxic small oligos bind synaptic receptors at sub-nanomolar affinities (9) and in so doing disrupt the intra-neuronal circuitry that underlies memory (8, 10) and foster tau fibril formation (11).

While the link between elevated cholesterol (CH) and increased AD incidence is well established (12, 13, 14, 15, 16, 17, 18), studies linking cholesterol 3-sulfate (CS) and AD are relatively scarce and have recently been reviewed (19). CS is synthesized from CH by sulfotransferase 2B1b and regulates several AD-relevant pathways. CS activation of the MINCLE receptor (20) leads to an elevated sterile-immune, pro-inflammatory response that enhances neurotoxicity in late-stage AD patients (21, 22). Further, CS allosteric activation of protein kinase C (23) causes hyper-phosphorylation and fibrillization of tau protein (24, 25).

CH (26, 27) and CS (28) dramatically accelerate Aβ-fibril formation, yet the acceleration mechanism remains largely undefined (28). Here, in a medley of experimentation and modeling, we quantitate the interactions of the ligands (CH and CS) with the Aβ_42_ peptide, define their effects on Aβ_42_-fibril formation, and construct molecular models that accurately describe the solution behavior of these systems and suggest a role for CS in senile plaque formation.

## Results and discussion

### Sterol binding to Aβ_42_

#### Equilibrium-binding studies

**Figure 1:**
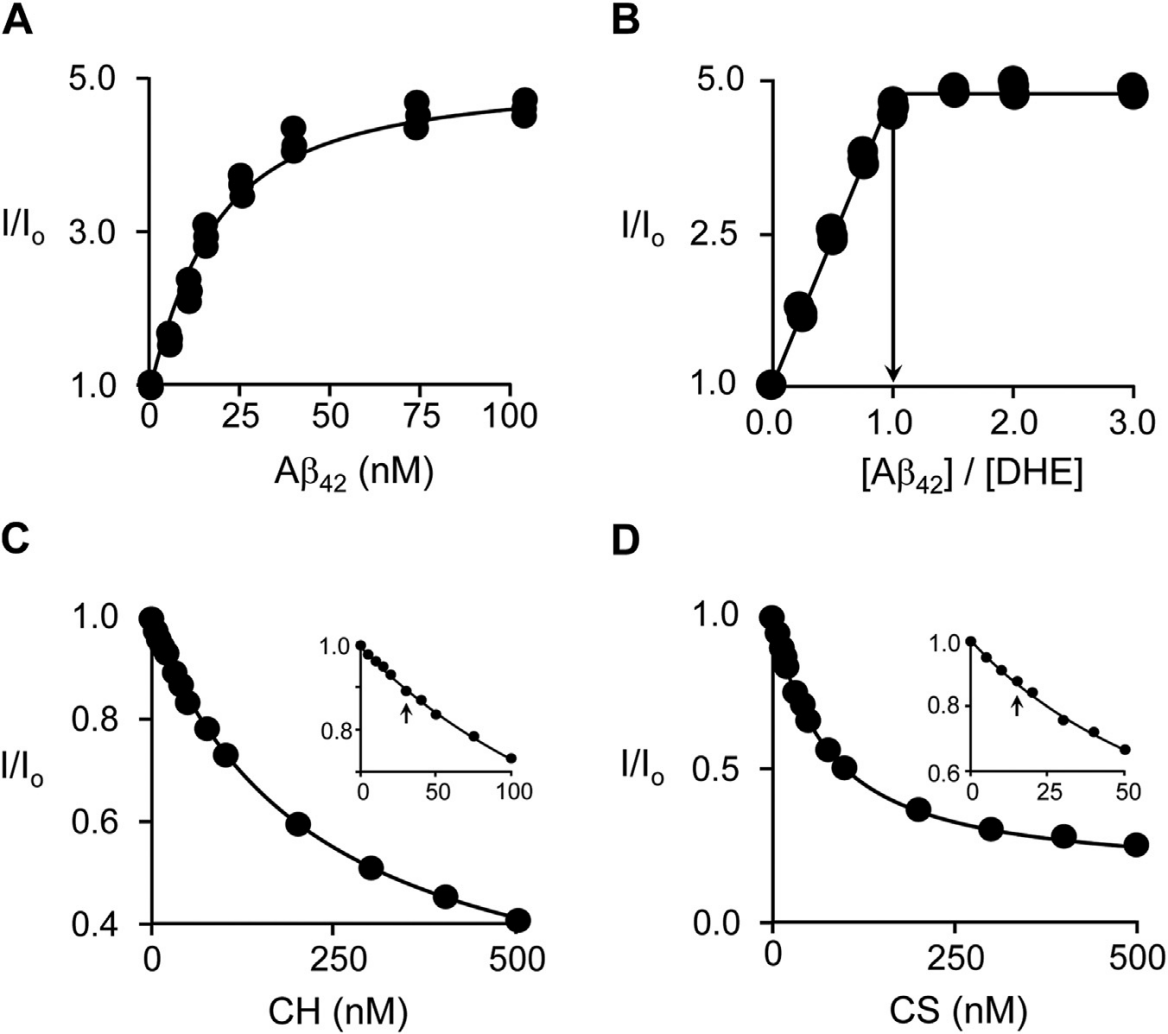
**Sterol binding to Aβ**_**42**_**at equilibrium.***A*, DHE affinity for monomeric Aβ4_2_. A solution containing DHE (10 nM, 0.83 × K_d_) and K_2_PO_4_ (50 mM), pH 7.4, 25 °C ± 2 deg. C, was titrated with Aβ_42_ (0–200 nM, 0–31 × K_d_). Binding was detected *via* the binding-induced 4.4 (±0.2)-fold increase in DHE fluorescence (λ_ex_ = 325 nm, λ_em_= 375 nm). Fluorescence intensity, I, is plotted relative to the intensity at zero titrant, I_0_. Titrations were performed independently in triplicate and the line passing through the data is the least-squares fit to a single-binding-site model. *B*, Aβ_42_:DHE stoichiometry. Aβ_42_ was titrated into to a solution containing DHE (fixed at 1.0 μM, 62 × K_d_) and K_2_PO_4_ (50 mM), pH 7.4, 25 °C ± 2 deg. C. DHE fluorescence (λ_ex_ = 325 nm, λ_em_ = 375 nm) is plotted *versus* [Aβ_42_]/[DHE]. The line descending from the titration breakpoint indicates a stoichiometry of 1:1. *C* and *D*, CH and CS affinities. Affinities were determined by competitive binding *versus* DHE. A solution containing DHE (10 nM, 0.67 × K_d_), Aβ_42_ (20 nM, 1.3 × K_d_), and K_2_PO_4_ (50 mM), pH 7.4, 25 °C ± 2 deg. C, was titrated with CH (5.0–500 nM, 0.05–5 × K_d_) or CS (5.0–500 nM, 0.15–33 × K_d_). Binding was monitored *via* the decrease in DHE fluorescence (λ_ex_ = 325, λ_em_ = 375) caused by DHE displacement from Aβ_42_. Controls ensured that <2.0% of Aβ_42_ oligomerized during the measurement. Titrations were performed in triplicate and averaged. The *solid lines* through the data represent least-squares fits to a competitive single-binding-site model. *C* and *D*, insets highlight the behavior of the titration over ligand concentrations that span their reported CMCs (indicated by *arrows*). K_d_ values are reported in Table 1.

CH (27) and CS (28) increase the rate of Aβ aggregation and CH is thought to stabilize fibrils (29); yet, the affinities of CH and its metabolites for the Aβ peptide monomer have not been determined. Here, CH and CS affinities for the Aβ_42_ monomer are established using dehydroergosterol (DHE)—a fluorescent CH analog (30) used as a competitive probe to determine CH and CS affinities. Aβ_42_ monomers were prepared as previously described (31) (see Methods). An Aβ_42_ titration of DHE is presented in Figure 1*A*. The titration data were least-squares fit to a single-binding-site model and the resulting best fit (indicated by the solid line) predicts a K_d_ of 16 ± 1 nM and a 4.4 ± 0.2-fold increase in DHE fluorescence at saturation. The stoichiometry of the Aβ_42_•DHE complex was determined in a second titration, Figure 1*B*, in which DHE is held fixed and saturated at 63 × K_d_ (1.0 μM). Under this condition, Aβ_42_ essentially quantitatively binds DHE until its concentration exceeds that of DHE, which results in a breakpoint in the titration curve that yields the stoichiometry. The arrow seen descending from the breakpoint indicates a stoichiometry of 1:1, which agrees well with the ∼1:1 CH-metabolite:Aβ stoichiometry estimated from analysis of senile plaques micro-dissected from AD-patient brain tissue (15).

CH and CS affinities were determined in competitive-binding studies in which DHE is progressively displaced by titration of the non-fluorescent ligand, Figure 1, *C* and *D*. CH and CS were titrated into an equilibrated solution containing Aβ_42_ (20 nM, 1.3 × K_d_) and DHE (10 nM, 0.67 × K_d_). Data were fit to a competitive single-binding-site model (32, 33, 34) (see Methods, Sterol binding to Aβ42 – Equilibrium studies). The best-fit results are shown as solid lines passing through the data and predict CS and CH dissociation constants of 36 ± 3 and 120 ± 10 nM, respectively (see Table 1).

**Table 1:** Sterol binding to Aβ_42_

Compound	K_d_ (nM)	K_d predicted_ (nM)^a^	k_o__ff_ (sec^−1^)	k_on_ (μM^−1^ sec^−1^)	k_off_/k_on_ (nM)
DHE	16 (1)^b^	21	1.0 (0.1)	60 (2)	17 (2.0)
CH	120^c^ (10)	160	2.5^d^ (0.1)	21^e^ (2)	120
CS	36^c^ (3)	31	0.88^d^ (0.05)	24^e^ (3)	36

a Molecular-dynamics predicted values.

b Parenthesises enclose one standard deviation.

c Determined in DHE competition studies.

d Determined in DHE displacement studies.

e Calculated by dividing k_off_ by K_d_.

Similar to other titrations involving CH (35, 36, 37), the CH and CS competition titrations (Fig. 1, *C* and *D*) are well-behaved and consistent with monomeric ligand. CH was reported in 1973 to form micelles with a 25 to 40 nM critical micelle concentration (CMC) (38); however, subsequent studies could not detect micelles at a concentration as high as 4.0 μM (39, 40, 41) and call into question the solution behavior of CH and its metabolites (41). The Figure 1, *C* and *D* insets highlight the titration regions that span the published CH and CS (28, 38) CMCs, which are indicated by vertical arrows. As evidenced by the excellent fits of the single-site binding model, the titrations predict the same K_d_ above and below the CMC; thus, CH and CS show no signs of aggregation throughout the titrations.

Aβ_42_ spontaneously forms fibrils, albeit slowly, in the absence of ligands, and polymerization is accelerated by CH and CS. It is thus important to establish that the ligand binding studies were performed on timescales short enough to avoid complications due to polymer formation. To do so, polymerization time-course controls (see Fig. S1) were run at the highest ligand and Aβ_42_ concentrations (*i.e.*, maximum oligomerization rates) associated with the Figure 1 titration. Polymerization was monitored using ThT, a fluorescent ligand widely used to detect Aβ_42_ oligomers ≥5-mers (42, 43). In the <30 min required to complete the Figure 1, *A* and *B* titrations, the control progress curves (Fig. S1, *A* and *B*) indicate ≤2% Aβ_42_ oligomerization. The Figure 1, *C* and *D* titrations required a shorter measurement time due to the acceleration of oligomerization caused by CS and CH (discussed below). The measurement time interval was reduced to <5 min by using freshly prepared solutions for each data point in the titration. Control progress curves (Fig. S1, *C* and *D*) indicate ≤2% Aβ_42_ oligomerized at the 5 min time-point.

#### Pre-steady state binding studies

**Figure 2:**
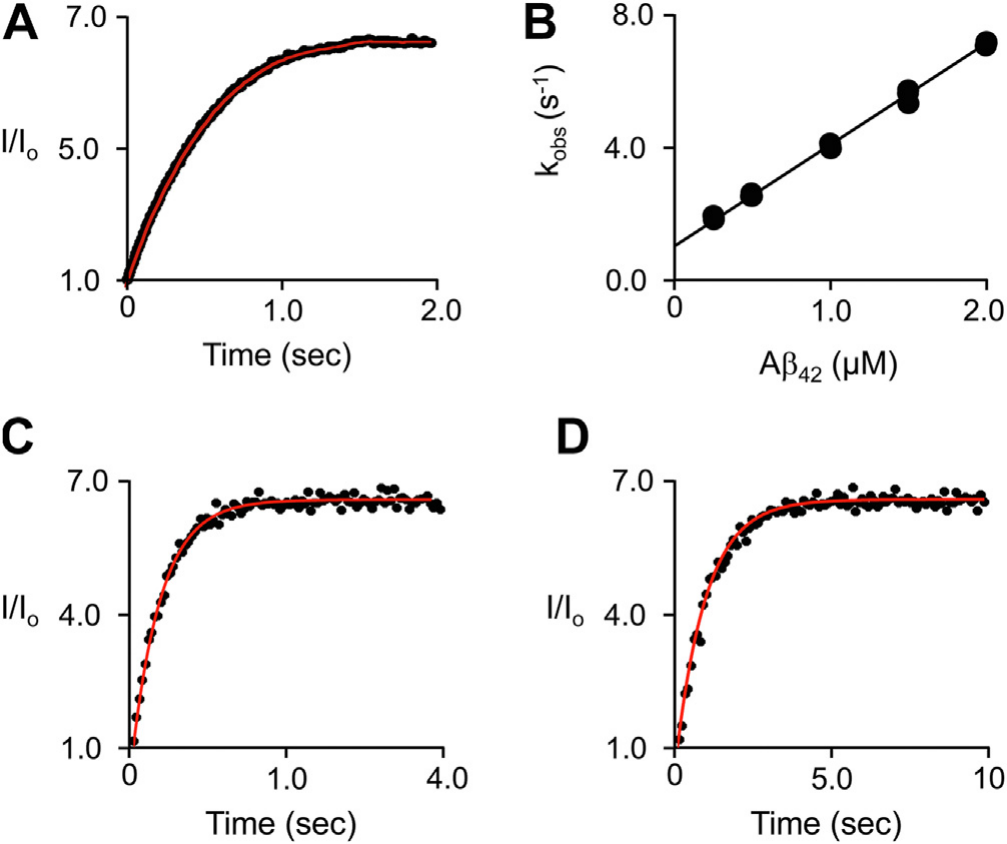
**Sterol binding to Aβ**_**42**_**—pre-steady state studies.***A*, DHE binding. Reactions were monitored *via* binding induced changes in DHE fluorescence using a stopped-flow fluorimeter (λ_ex_ = 325, λ_em_ > 400 nm). Fluorescence intensity, I, is reported relative to the intensity in the absence of ligand, I_o_. A solution containing Aβ_42_ (2.0 μM, 74 × K_d_), K_2_PO_4_ (50 mM), pH 7.4, 25 °C ± 2 deg. C was rapidly mixed (1:1, v/v) with a solution identical except that Aβ_42_ was replaced by DHE (40 nM, 2.7 × K_d_). The bindingreaction curve shown is the average of five independent progress curves. The averaged curve was a least-squares fit to a single-exponential equation and the resulting best fit (indicated by the *red line*) yielded k_obs_. *B*, DHE k_on_ and k_off_. *k*_*obs*_ values obtained as in Panel *A* were determined in triplicate at varying Aβ_42_ concentrations and the averaged values are shown plotted *versus* [Aβ_42_]. k_on_ and k_off_ are given by the slope and intercept of a linear least-squares fit of the k_obs_*versus* [Aβ_42_] plot. *C* and *D*, CH and CS displacement reactions. A solution containing DHE (10 μM, 630 × K_d_), K_2_PO_4_ (50 mM), pH 7.5, 25 °C ± 2 deg. C was rapidly mixed (1:1, v/v) with a solution in which DHE was replaced with Aβ_42_ (0.10 μM, 0.83 × K_d_) and CH (2.0 μM, 17 × K_d_), Panel *C*, or, Aβ_42_ (0.10 μM, 2.9 × K_d_) and CS (0.60 μM, 17 × K_d_), Panel *D*. The final DHE concentration (5.0 μM, 330 × K_d_) displaces ∼98 % of either CH (1.0 μM, 8.3 × K_d_) or CS (0.30 μM, 8.3 × K_d_); hence, the dissociation reactions are pseudo first order. k_off_ was obtained from a least-squares fit to a single-exponential equation (shown as *red line* passing through the data).

To establish the rates at which Aβ_42_•ligand complexes form and dissociate, the rate constants governing the binding reactions were determined. DHE binding was monitored in real time using a stopped-flow fluorimeter *via* binding-induced changes in DHE fluorescence. A representative binding-reaction progress curve is presented in Figure 2*A*. The reactions are pseudo first order in [ligand] and k_obs_ was obtained by least-squares fitting progress curves to a single-exponential equation (44, 45). k_obs_ values were determined (in triplicate) at a series of Aβ_42_ concentrations and the averaged values are plotted *versus* [Aβ_42_] in Figure 2*B*. k_on_ and k_off_ are given by the slope and intercept of the k_obs_-*versus*-[Aβ_42_] plot obtained by linear least-square fitting (44, 45). CH and CS k_off_ values were determined in competitive stopped-flow fluorescence studies in which CH or CS (8.5 × K_d_, post mixing) is ∼98% displaced by DHE (330 × K_d_, post mixing) — Figure 2, *C* and *D*. Under these conditions, the displacement reactions are irreversible first-order and k_off_ values are obtained by least-squares fitting to a single-exponential equation. k_on_ values were calculated using k_off_ and the corresponding K_eq_ values. The rate constants are compiled in Table 1. The monophasic nature of the DHE-binding reactions is consistent with binding to a single form of Aβ_42_ and the coincidence of k_off_/k_on_ (17 ± 2 nM) and K_d_ (16 ± 1 nM) suggests that equilibrium and pre-steady state measurements monitor interactions among the same species. The on-rate constants for formation of all three complexes range from 2 to 6 × 10^7^ M^−1^ s^−1^ and are close to the value calculated for the diffusion encounter of DHE and Aβ_42_, 9.3 × 10^7^ M^−1^ s^−1^, using the DHE diffusion constants and Stokes radii (46, 47) and Aβ_42_ (48, 49); hence, components of the complexes exchange rapidly and, as will become apparent, complex formation and dissociation does not contribute meaningfully to the rate of Aβ_42_-oligomer formation.

#### CH and CS accelerate Aβ_42_ nucleation

**Figure 3:**
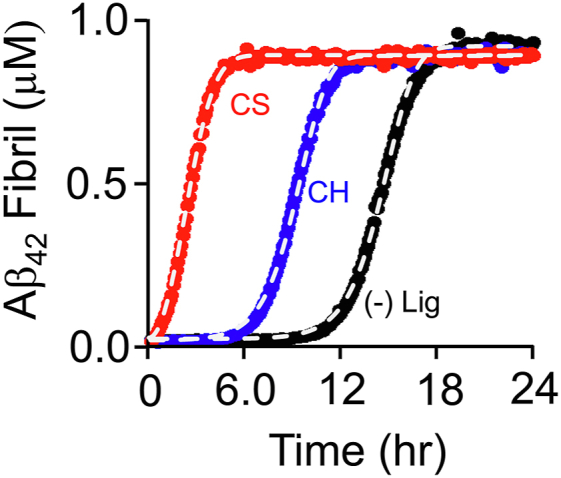
**Formation of CH•, CS•, and unliganded Aβ**_**42**_**fibrils.***A*, fibril formation. Fibril formation was monitored *via* the fluorescence increase (λ_ex_ = 450 nm, λ_em_ = 482 nm) associated with the binding of ThT, which binds Aβ oligomers ≥5-mers. Reaction conditions: Aβ_42_ (1.0 μM), ThT (30 μM), ligand (CH (3.0 μM, 17 × K_d_) *or* CS (1.6 μM, 17 × K_d_) *or* no ligand), DMSO (0.5 % v/v), K_2_PO_4_ (50 mM), pH 7.4, 25 °C ± 2 deg. C. Progress curves were performed in triplicate, averaged, and fit numerically using the NEF polymerization model (see Results and discussion). Lines passing through the data represent the best fits of the NEF model and the associated rate constants are listed in Table 2.

The effects of CS and CH on Aβ_42_ oligomerization were assessed by monitoring fibril formation in the presence and absence of ligands using ThT — a fluorescent sensor that binds Aβ oligos ≥5-mers (42, 43) and does not perturb polymerization progress curves at concentrations below 50 μM (50). Reactions were initiated by the addition of ligand (at saturation) or buffer (control) to a fresh solution of Aβ_42_ and ThT (see Fig. 3 Legend). Reactions were performed in triplicate and the averaged data are presented in Figure 3. Such progress curves are often fit using a generalized sigmoidal function and compared on the basis of lag-time length and elongation-phase slope. Here, progress curves are fit numerically to the Nucleation-Elongation-Fragmentation (NEF) model (51, 52), using *Copasi* (53). Numerical modeling allows one to assess whether a particular mechanism adequately predicts experimental behavior and provides rate constants for the steps of the mechanism that can be compared across peptide•ligand combinations to identify where and to what extent ligands induce change.

The NEF model assumes a slow, energetically unfavorable event generates a polymerization nucleus that rapidly adds monomers (at the elongation rate) to form oligomers. The reaction accelerates in a geometric fashion as oligomer fragmentation produces new nuclei. The tenets of the model are embodied in Equations (1), (2), (3), which form the basis of the *Copasi* model (53).

(1) $$2m\;\;\overset{k_{nuc\;for}}{\underset{k_{nuc\;rev}}{⇆}}\;n$$

(2) $$m+n\;\overset{k_{el\;for}}{\underset{k_{el\;rev}}{⇆}}\;o+n$$

(3) $$o\underset{k_{frag}}{\to }\;n$$

where *m* is monomer concentration, *n* is the concentration of surfaces capable of adding monomer, and *o* is the concentration of peptides that are in the interior of the polymer and thus cannot add monomers. *k*_*nuc*_, *k*_*el*_, and *k*_*frag*_ are the nucleation, elongation, and fragmentation rate constants.

**Figure 4:**
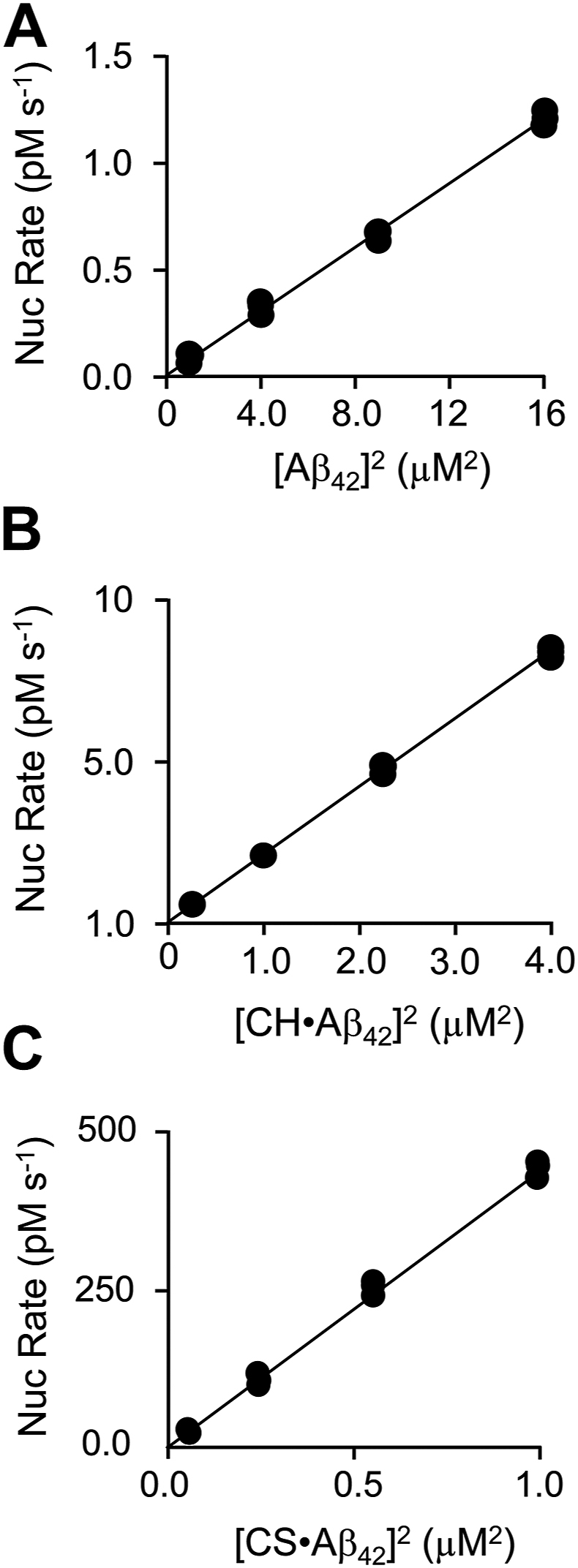
**Nucleation reaction order.** Reactions were monitored *via* binding-induced changes in ThT fluorescence (λ_ex_ = 450 nm, λ_em_ = 482 nm). All reactions were performed under the following conditions: ThT (30 μM), DMSO (0.5 % v/v), and K_2_PO_4_ (50 mM), pH 7.4, 25 °C ± 2 deg. C. Ligand concentrations were adjusted to maintain free [ligand] at 17 × K_d_. *A*, unliganded Aβ_42_ nucleation. Titrant concentrations: Aβ_42_ (1.0, 2.0, 3.0, or 4.0 μM). *B*, CH•Aβ_42_ nucleation. Titrant concentrations: Aβ_42_ (0.5, 1.0, 1.5, or 2.0 μM) and CH ([Aβ_42_] + 17 × K_d_; 2.5, 3.0, 3.5, and 4.0 μM). *C*, CS•Aβ_42_ nucleation. Titrant concentrations: Aβ_42_ (0.25, 0.50, 0.75, 1.0 μM) and CS ([Aβ_42_] + 17 × K_d_; 0.85, 1.1, 1.35, and 1.60 μM). Reactions were run in triplicate and plotted *versus* [Aβ_42_]^2^. Nucleation rate constants are given by the slope of linear least-squares fits shown as lines passing through the data.

Reaction 1 of the NEF scheme assumes nucleation is second order in Aβ_42_ monomer concentration. The reaction order was determined for ligand-bound and free forms of Aβ_42_ by plotting nucleation rates *versus* [Aβ_42_]^n^, where *n*, the reaction order, is 1, 2, or 3—such plots become linear only when *n* equals the reaction order (54). Nucleus formation was monitored *via* ThT fluorescence in the region of the progress curve where nucleation is rate limiting—the very early stage of the lag phase. Rates were obtained by linear least-squares fitting of progress curves. In all cases, fits were initiated at t_0_ and deviation from linearity was within experimental error (*i.e.*, χ^2^ > 0.95). As is evident in Figure 4, *A*–*C*, the Rate-*versus*-[Aβ_42_]^n^ plots for all three peptide forms are linearized at n = 2; hence, the nucleation reactions are second order. The nucleation rate constants are given by the slopes of the plots (obtained by linear least-squares fitting) and are within the error of those predicted by NEF fitting, see Table 2.

**Table 2:** Aβ_42_ Fibril formation rate constants

Ligand	Nucleation (nM^−1^ s^−1^)		Fragmentation (s^−1^)^a^	Elongation^a^	
	Linear model^b^	NEF model^a^		Forward (μM^−1^ s^−1^)	Reverse (s^−1^)
None	1.6 (0.1)^c^ × 10^−15^	1.4 (0.2)^b^ × 10^−15^	3.5 (0.2) × 10^−11^	660 (20)	70 (2)
CH	7.3 (0.1) × 10^−14^	6.9 (0.2) × 10^−14^	5.6 (0.3) × 10^−11^	830 (20)	98 (1)
CS	1.5 (0.1) × 10^−11^	1.8 (0.3) × 10^−11^	4.3 (0.7) × 10^−11^	860 (60)	85 (1)

a Obtained from NEF fitting fibril formation progress curves (Fig. 3).

b Obtained from linear least-squares fitting *Rate-versus-[Aβ*_*42*_*]*^*2*^ data (Fig. 4, *A*–*C*).

c Parentheses enclose one standard-deviation unit.

The polymerization progress curves seen in Figure 3 indicate similar acceleration and elongation phases for all three curves (Aβ_42_, Aβ_42_•CH and Aβ_42_•CS). The NEF fits are shown as solid lines passing through the data and the associated rate constants are listed in Table 2. The NEF model predicts forward and reverse elongation rate constants that are within 1.3- and 1.4-fold of one another, respectively, and that fragmentation rate constants differ less than 1.6-fold. In contrast, the forward nucleation rate constants differ dramatically. Relative to the unliganded peptide, the nucleation rate constant is increased 49-fold by CH, and, remarkably, ∼13,000-fold by CS — a profound acceleration of the rate at which the nuclei that polymerize fibril synthesis are produced.

### Molecular dynamics analysis

To establish molecular mechanisms capable of predicting Aβ_42_ behavior in the presence and absence of ligands, molecular dynamics models were developed using GROMACS (55, 56). The accuracy of the models was vetted by comparing their predictions with experimental outcomes.

#### Aβ_42_ monomers

Simulations utilized a 5.0 × 5.0 × 5.0 nm cube containing water, PO_4_ (50 mM) and KCl (0.10 M), pH 7.4, 25 °C. To minimize docking bias, Aβ_42_ and ligands were semi-randomly positioned in the cube such that their inter-atom distances were ≥5 Å.

##### Unliganded peptide

To identify the peptide form to use in ligand binding studies, the dual-helix Aβ_42_ structure (57) often used in MD simulations (58, 59, 60) was equilibrated over 5.0 μs. Consistent with NMR modeling (61, 62, 63), the helices unwound (t_1/2_ = 1.2 μs) into a persistent random coil, which was used as the initial unliganded Aβ_42_ configuration in all subsequent simulations.

##### CH•peptide

**Figure 5:**
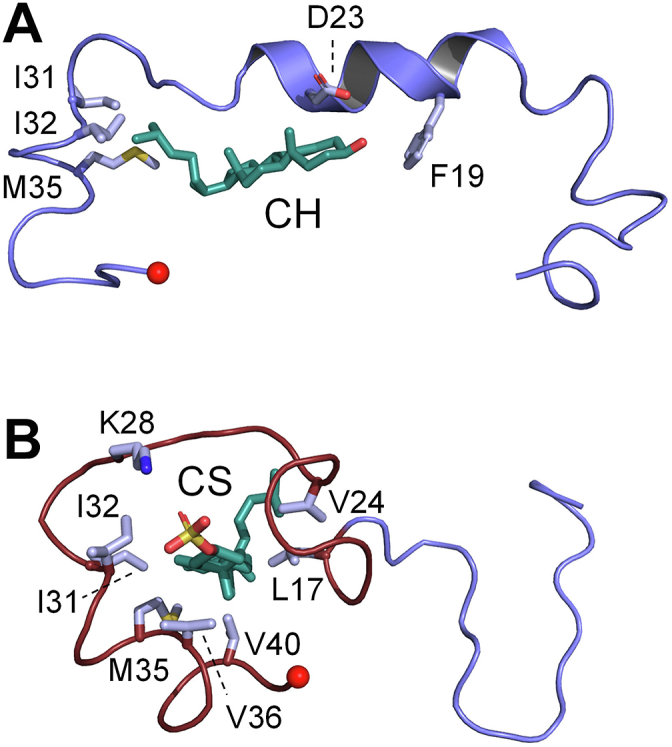
**MD-predicted Aβ**_**42**_**monomer structures.***A*, the CH•Aβ_42_ monomer. *B*, the CS•Aβ_42_ monomer. All residues in direct contact with the ligand are shown in “*stick*” and labeled. *Small red spheres* mark the Aβ_42_ peptide C-terminal residue, A42.

CH and Aβ_42_ were allowed to freely associate over 1.0 μs. Cluster analysis, which groups structures according to their RMSD similarity (<5 Å cutoff), identified the six CH•Aβ_42_ forms seen in Fig. S2. The predominant form, Form 1 (Fig. 5*A*), is present during 74% of the simulation; the remaining forms and their percent presence are as follows: 2 (8.7%), 3 (6.8%), 4 (5.2%), 5 (3.3%), 6 (1.7%). Notably, only Form 3 is devoid of α-helical structure and, as described below, is the only complex predicted to form dimers. The K_d_ predicted for the CH•Aβ_42_ complex, 160 nM, agrees well with the experimentally determined value of 120 ± 12 nM (see Table 2).

##### CS•peptide

The predicted structure of the CS complex, Figure 5*B*, is distinct from the CH-bound species — the peptide is devoid of α-helix and encircles the central aspect of the CS carbon skeleton in a hydrophobic ring (rendered in brown). The CS sulfate moiety, which extends beyond the ring, forms a salt bridge with the K28 primary amine. Cluster analysis indicates that the ring is present during >98% of the simulation and the peptide N-terminus behaves as a random coil. The predicted CS•peptide dissociation constant, 31 nM, is in excellent agreement with the experimentally determined value, 36 ± 3 nM (see Table 2).

#### Aβ_42_ dimers (polymerization nuclei)

**Figure 6:**
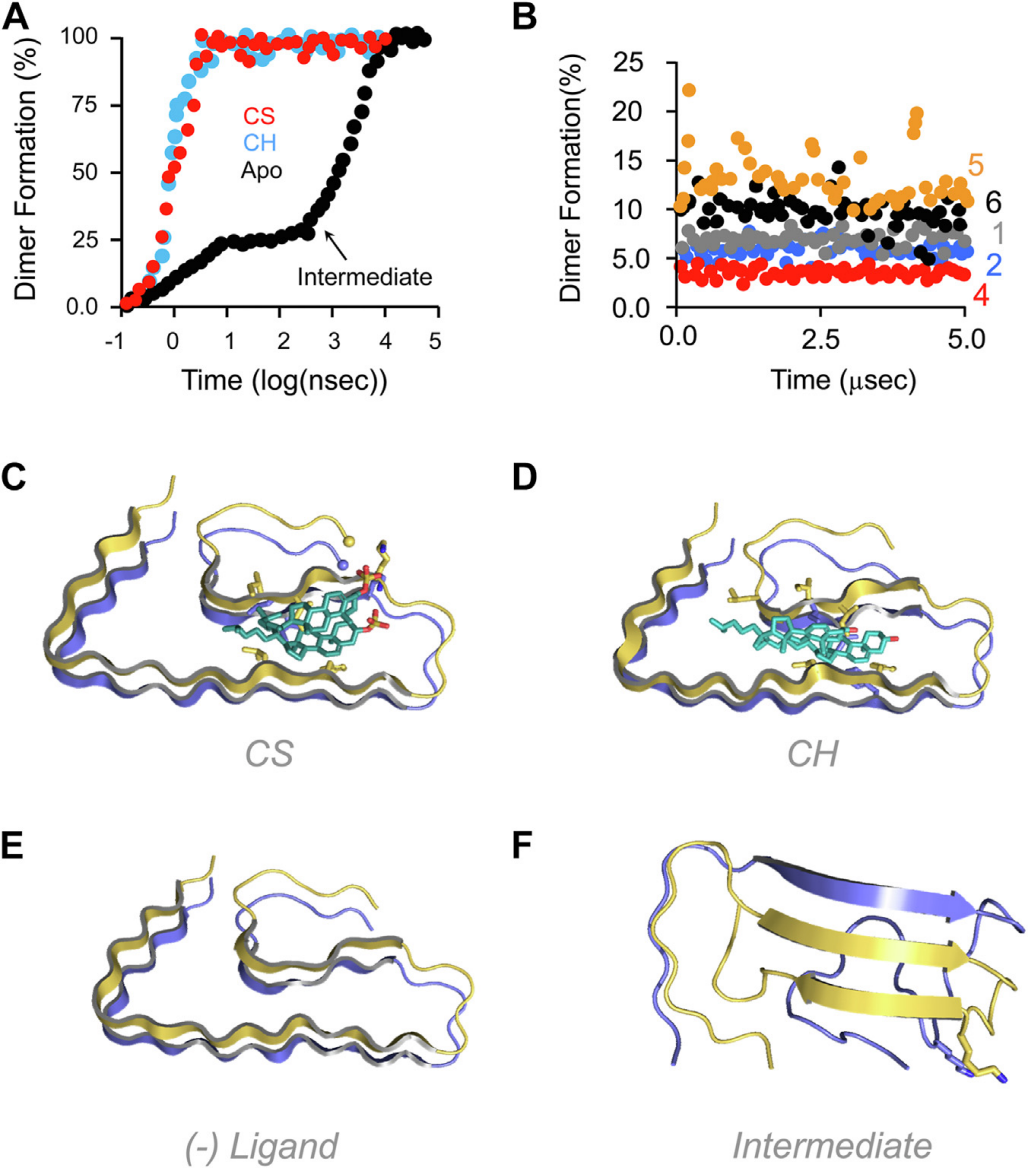
**Dimerization studies.***A* and *B*, dimerization is monomer dependent. Eight monomer forms (unliganded peptide, CH•peptide Forms 1–6, and CS•peptide) were tested in MD simulations for their tendency to form dimers over a 5.0 μs time interval. Panel *A* presents progress curves for the species that formed dimers — *i.e.*, CS•peptide (CS), Form 3 CH•peptide (CH), and unliganded peptide. Panel *B* shows the progress curves for CH Forms that did not yield dimers. The curves are numbered according to the CH Forms given in Fig. S2. *C–F*, dimer structures. Panels *C–E* present the time average of the structures in the plateaus of the CS•, CH•, and unliganded-peptide progress curves, respectively. Panel *F* presents the structure of the unliganded-peptide intermediate that rapidly forms and slowly rearranges to the structure seen in Panel *E*.

Each of the eight peptide forms—six CH, one CS, and the unliganded form—was tested for its tendency to dimerize by randomly positioning two copies of a given species inside an aqueous cube and monitoring dimer formation *versus* time over 5.0 μs (2 ps step-size). CH monomers were constrained to remain within their clusters by a weak restoring force (56) until significant inter-peptide contact was established at which point the constraint was removed and monomers could reconfigure freely. Significant contact is defined as an interaction energy exceeding ∼10% of the energy required to separate two fully associated peptides (25 kJ/mole (64)). In Figure 6, *A* and *B*, the dimerization reactions are grouped according to those that either did or did not produce dimers, respectively. The grouping reveals that only monomers devoid of α-helical structure form dimers—the Form 3 CH, CS, and unliganded species. The time average of all structures in the dimerization plateaus is shown in Figure 6, *C*–*E*. The structures are highly similar, ∼79% β-sheet, and fully consistent with the S-shaped peptide structure seen in fibrils (65).

Unliganded Aβ_42_ forms the S-shaped dimer found in fibrils slowly relative to the liganded peptide (Fig. 6*A*). Structural “snapshots” along the reaction coordinate reveal that unliganded Aβ_42_ rapidly (∼3 ns) forms a single stable intermediate—a dimer containing three parallel β-sheets (Fig. 6*F*) that slowly transitions to the more stable S-shaped β-sheet structure seen in fibrils. The half-life for transitioning the intermediate to the S-shaped dimer, 6.0 μs, is 11,300-fold greater than that for converting CS monomers to dimers, 0.53 ns—a value that compares favorably with the 13,000-fold value determined experimentally (see Table 2). Thus, the model predicts that CH and CS accelerate dimerization relative to the unliganded species by “guiding” the peptide folding reaction away from the intermediate.

To test the MD prediction that only ligand-bound monomers devoid of α-helix readily form dimers, theoretical and experimental dimerization rates were compared. Modelling indicates ∼100% of CS and 6.8% of Form 3 CH monomers are dimerization competent. Dimerization is proportional to the square of the monomer concentration; thus, the predicted CS/CH dimerization rate ratio is (1.0/0.068)^2^ = 216. The experimental ratio, calculated using nucleation rate constants (see Table 2) is 261-fold. The close agreement of these values lends credence to the model and its underlying mechanism. It is notable that CS and Form 3 CH monomers form homo- and heterodimers at indistinguishable rates (see Fig. S3).

#### The Aβ_42_ oligomer

**Figure 7:**
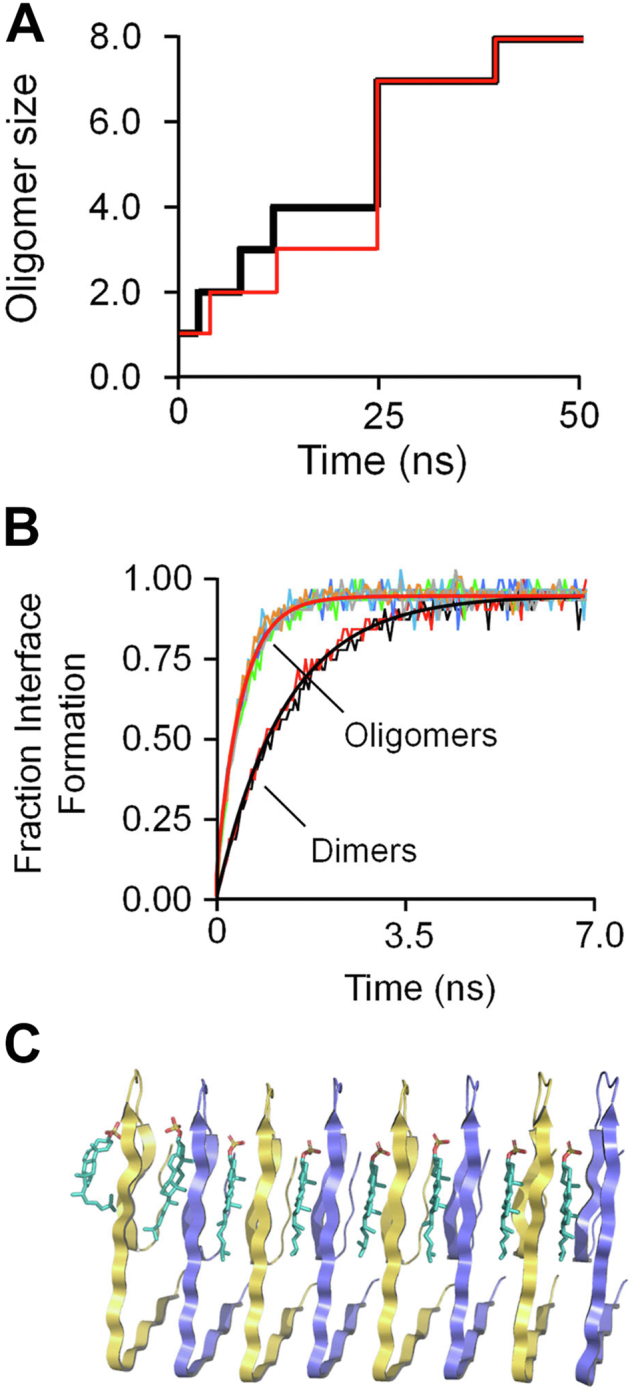
**CS•Aβ**_**42**_**oligomerization studies.***A*, CS•Aβ_42_ octamer assembly. Eight CS•Aβ_42_ monomers are seen spontaneously assembling into an octamer. *Black* and *red lines* trace the oligomerization status of the two peptides that initiate oligomerization. The simulation was initiated with eight monomers randomly positioned in a 10 × 10 × 10 nm cube of water, PO_4_ (50 mM), KCl (0.10 mM), pH 7.4, 25 °C. *B*, interface formation. Each progress curve shows the transition of two interface-forming species from an early-contact stage to a fully formed interface. The seven transitions associated with octamer assembly are included in the figure. The curves are separated based on reaction rate into two classes — dimer interface formation, and oligomer interface formation (which includes monomer/oligo and oligo/oligo interfaces). The progress curves in each class were least-squares fit to a single exponential equation, the best-fit k_obs_ values within each class were averaged and the average value was used to generate the solid lines seen passing through the datasets. *C*, predicted structure of the CS octamer.

A time course for CS•Aβ_42_ octamer formation is presented in Figure 7*A*, which plots the oligomerization status of two CS•peptides *versus* time. The first peptide, associated with the black line (*BL*), forms a dimer at ∼6 ns; the second, associated with the red-line (*RL*), forms a dimer ∼2 ns later. The BL dimer forms a trimer and then tetramer before combining with the RL-associated trimer, which formed in the interim, to produce a 7-mer that then adds the final monomer to produce the octamer. The formation of new peptide interfaces, which appear as vertical lines, is extremely fast relative to the peptide-encounter time intervals, which lengthen as the concentration of interacting species decreases. Progress curves for the interface-forming reactions, seen in Figure 7*B*, cluster into two groups—those that form dimers and those that add to an existing oligomer, which unlike the monomer presents a preformed β-sheet structure to the incoming species. The k_obs_ values associated with dimer- and oligomer-interface formation are 0.76 ± 0.01 ns^−1^ and 2.2 ± 0.03 ns^−1^, respectively; hence, the preformed β-sheet scaffold accelerates interface formation 2.9-fold. The structure of the self-assembled octamer, depicted in Figure 6*C*, is fully consistent with the cryo-EM structure (66).

#### Oligomer fragmentation

Fragmentation, a cardinal feature of the NEF model, transitions fibril formation from the nucleus-forming, or lag phase, to the extension phase by the geometric expansion of the number of oligos undergoing polymerization (51, 52, 53). The molecular dynamics model was tested for its ability to fragment oligomers by placing CS•oligomers (8-mer, 16-mer or 25-mer) in 10 × 10 × 10 nm cube containing water, PO_4_ (50 mM), KCl (0.10 M), pH 7.4, 298 K, and monitoring oligomer size over a 0.10 μs time interval. While neither the 8- nor 16-mer fragmented, the 25-mer was cleaved in a nearly simultaneous double-fragmentation event into three oligos, a 7- eight- and 10-mer (see Fig. S4).

#### Biological inferences

Given that the nucleation rate constant is 260-fold greater for CS•Aβ_42_ than CH•Aβ_42_, it is of interest to consider whether CS might meaningfully contribute to nuclei synthesis *in vivo*. The levels of CS in adult human brain have not been published; however, CH (67) and CS (68) levels in normal rat brain predict CS/CH ratios that range from ∼0.05 to 2.5% (67, 68). The CH level in human cerebrospinal fluid (∼6.5 μM) (69) is quite high relative to its Aβ_42_ affinity (120 nM) and suggests CH is saturating *in vivo* — a perspective supported by the fact that plaques isolated from human brain are saturated in with sterols (primarily CH) in a 1-to-1 stoichiometry (15). Assuming CH and CS are saturating, and setting CS at 1% of CH on a molar basis, 3.4% of Aβ_42_ is expected to reside in the CS•Aβ_42_ complex due to the 3.4-fold enhanced affinity of CS over CH for Aβ_42_. The nucleation rate constants in Table 2 predict that at 3.4% CS•Aβ_42_, CS homodimer nuclei will form at ∼one-third the rate of CH homodimers. Given that 100% of CS and 6.8% of CH monomers are dimerization competent, and that the rate constants for homo- and heterodimer formation are identical (Fig. S3), the CS/CH heterodimers will form at 64% the rate of CH homodimers. Together these values predict that 49% of polymerization nuclei will contain one or more CS.

The role of CS in small Aβ-oligo toxicity has not been considered. Neurotoxic Aβ oligos are ∼12 monomers in length (8). Nuclei lengthen *via* the addition of CH and CS monomers in their solution ratio; hence, the CS composition of a given length oligo can be calculated. Again assuming CS is 1% of CH on a molar basis, one calculates that in addition to the CS composition of the nucleus, ∼30% of 12-mers will contain one or more additional CS and ∼8% will contain two or more — for example, 30% of 12-mers initiated with CS homodimers will contain three or more CS, and ∼8% will contain four or more.

**Figure 8:**
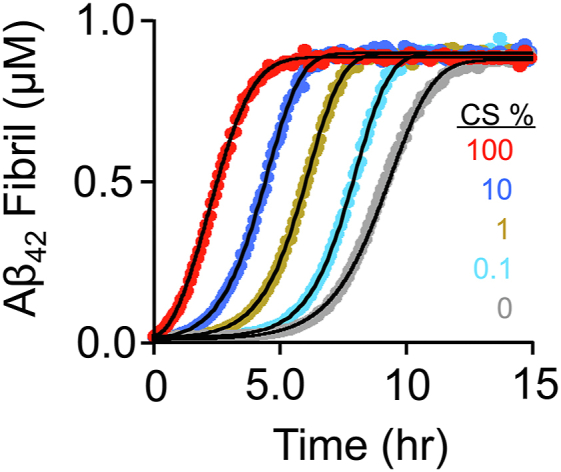
**Aβ**_**42**_**fibril formation *versus* CS percentage.** Fibril formation was detected *via* binding-induced changes in ThT fluorescence (λ_ex_ = 450 nm, λ_em_ = 482 nm). Reaction conditions: Aβ_42_ (1.0 μM), CS percent (as indicated), [CS] + [CH] (20 μM), ThT (30 μM), DMSO (0.5 % v/v), K_2_PO_4_ (50 mM), pH 7.4, 25 °C ± 2 deg. C. Progress curves were performed in triplicate and averaged data are shown. Lines passing through the data represent the behavior predicted by the NEF model using the Table 2 rate constants.

To experimentally assess the potential impact of CS on fibril formation *in vivo*, fibril synthesis was monitored over a series of CS/CH ratios that span the physiological range. As is apparent in Figure 8, at 0.1% CS — the level in human blood (70) — the lag-time shortens by several hours; at 1% — the level in rat brain tissue (67, 68) — the lag-time is nearly halved; and at 10% — the level in human skin (71) — the lag-time approaches that of pure CS.

The extent to which CS-accelerated nucleation contributes to the rate of plaque formation in the AD-diseased brain is not known. Should its role be significant *in vivo*, it is most likely to contribute in the earliest stages of the disease, perhaps prior to plaque detection, where the contribution from fibril fragmentation is at a minimum.

## Conclusions

The rate constants and equilibria governing Aβ_42_ monomer interactions with CH and CS were determined and reveal that Aβ_42_•ligand complexes form and dissociate rapidly relative to Aβ-fibril formation. Ligand induced stimulation of fibril formation is shown to occur nearly exclusively in the nucleation phase of the reaction. Relative to the unliganded peptide, nucleation is accelerated 49-fold by CH, and, remarkably, 13,000-fold by CS. Molecular dynamics models that accurately predict peptide/ligand affinities and rate accelerations offer atomic/molecular descriptions of the fibril formation mechanism. Modeling predicts that the experimentally observed 260-fold difference in CH- and CS-induced nucleation rates are due to ligand-dependent differences in the dimerization competency of the monomers — 100% *versus* 6.8% for CS- and CH-bound species — and that competency is determined by the extent to which the ligand-bound species are devoid of α-helix. Relative to the unliganded peptide, CH and CS accelerate dimerization primarily by guiding the peptide folding trajectory away from a non-productive β-sheet-rich intermediate that profoundly slows its transition to a polymerization-competent dimer. Finally, experiments and modeling over presumed physiological CS/CH ranges imply that CS - might well contribute to senile plaque accumulation in the AD patient brain.

## Experimental procedures

### Materials

Amyloid-β peptide (>96% pure), 1,1,1,3,3,3-hexafluoro-2-propanol (HIFP, >99% pure) and potassium phosphate (ACS grade) were obtained from the Sigma-Aldrich Corp. Ampicillin, dimethyl sulfoxide (DMSO), dehydroergosterol (DHE), and KOH, were purchased from Fisher Scientific. Cholesterol (CH) and cholesterol sulfate (CS) were purchased from Avanti Polar Lipids, Inc. Simulations were performed on a QS32 to 2670C-XS8 Parallel Quantum Solutions computer. GROMACS and GROMASS57 energy fields are available under a General Public License (56). SWISS MODELLER is freely available from the Swiss Institute of Bioinformatics (72). Automated Topology Builder (ATB), an energy parametrization algorithm, is maintained by the National Computational Infrastructure at Australia National University (https://atb.uq.edu.au/ (73)). PyMol was purchased from Schrödinger.

### Methods

#### Preparation of Aβ_42_ monomers

Aβ_42_ monomers were prepared using a well-established method (31). Briefly, peptide is resuspended in HIFP, vortexed 30 s to form stable α-helical monomers (57), and aliquoted (50 μg/tube) into 0.5 ml Eppendorf tubes. HIFP is then evaporated (ambient temperature) in a fume hood overnight and the remaining HIFP is removed under vacuum (1 h) without heating using a SpeedVac. Samples tubes are then sealed with paraffin and stored at −20 °C. Immediately prior to use, the peptide-containing tubes are warmed to room temperature and DMSO is added to create a 5.0 mM peptide stock solution that is added as needed to experimental buffers. The excess peptide is discarded after 8 h.

#### Sterol binding to Aβ_42_ – Equilibrium studies

DHE/Aβ_42_ binding interactions were monitored *via* the binding-induced 4.4-fold change in DHE fluorescence (λ_ex_ = 325 nm, λ_em_= 375 nm). Solutions containing DHE (10 nM, 0.83 × K_d_), K_2_PO_4_ (50 mM), pH 7.4, 25 °C ± 2 deg. C were titrated with Aβ_42_ (0–500 nM, 0–31 × K_d_). Independent triplicate titrations were performed and K_d_ was obtained by least-squares fitting the bound fraction DHE (F_DHE_) to a single-binding-site model (Equation 4) (32, 33, 45).

(4) $$F_{DHE}=\frac{\left(\left[\text{DHE}\right]+\left[{A\beta }_{42}\right]+K_{d}\right)-\sqrt{{\left(\left[\text{DHE}\right]+\left[{A\beta }_{42}\right]+K_{d}\right)}^{2}-4\times \left[{A\beta }_{42}\right]\times \left[\text{DHE}\right]}}{2\times \left[DHE\right]}$$

The DHE:Aβ_42_ binding stoichiometry was determined by titrating Aβ_42_ (0–3.0 μM, 0–186 × K_d_) into a solution containing DHE (1.0 μM, 62 × K_d_) and K_2_PO_4_ (50 mM), pH 7.4, 25 °C ± 2 deg. C. The high-saturating DHE concentration causes a sharp break point in the titration that yields the binding stoichiometry (see Results and discussion). The dilution caused by titrant addition (<5% overall) was taken into account in data analysis.

The affinities of CH and CS for Aβ_42_ were determined in competitive binding studies *versus* DHE. Binding was detected *via* the fluorescence decrease (λ_ex_= 325 nm, λ_em_ = 375 nm) caused by DHE displacement. Titration solutions contained DHE (10 nM, 0.67 × K_d_), Aβ_42_ (20 nM, 1.3 × K_d_), CH (5.0–500 nM, 0.05–5 × K_d_) or CS (5.0–500 nM, 0.15–33 × K_d_), K_2_PO_4_ (50 mM), pH 7.4, 25 °C ± 2 deg. C. CS and CH concentrations spanned their reported CMCs (28, 38). Measurements were made within 5 min post-mixing to ensure <2% of Aβ_42_ oligomerized during the measurements. As described previously (74), data were fit to the following competitive single-site-binding model equation (Equation 5):

(5) $$\frac{I}{I_{0}}=\frac{C+I_{\max}\times \left[L\right]}{\left[L\right]+C\times K_{d\;L}}$$

where C = 1 + ([DHE]/K_d__DHE_), [L] and K_d L_ are concentrations and affinities associated with CS or CH, and I_max_ is the fluorescence change associated with displacement of all peptide-bound DHE. I_max_ = (fraction DHE-bound to peptide at zero competitive ligands)/4.4 — the fold fluorescence change associated with DHE dissociation, see Figure 1*A*.

#### Sterol binding to Aβ_42_—Pre-steady state studies

Pre-steady-state binding of sterols to Aβ_42_ was monitored *via* the binding-induced change in DHE fluorescence (λ_ex_ = 325, λ_em_ > 400 nm) using an Applied Photophysics SX20 stopped-flow fluorimeter.

##### DHE binding

Progress curves were obtained by rapidly mixing (1:1 v/v) a solution containing DHE (0.50 μM, 33 × K_d_) and K_2_PO_4_ (50 mM), pH 7.4, 25 °C ± 2 deg. C, with a solution that was identical except that it contained Aβ_42_ (0.30–4.0 μM, 20–260 × K_d_). Three progress curves (each an average of five independently obtained curves) were collected at five separate Aꞵ_42_ concentrations. The observed rate constant (k_obs_) at a given [Aβ_42_] was obtained by fitting the average of the three curves to a single-exponential equation using Pro-K software (75). k_on_ and k_off_ were obtained from the slopes and intercepts predicted by linear least-squares analysis of k_obs_ *versus* [Aβ_42_] plots.

##### CH and CS binding

Progress curves were obtained by rapidly mixing (1:1 v/v) a solution containing Aβ_42_ (10 nM), CH (2.0 μM, 16 × K_d_) or CS (600 nM, 17 × K_d_), K_2_PO_4_ (50 mM), pH 7.4, 25 °C ± 2 deg. C, with a solution containing DHE (2.5 μM, 160 × K_d_), K_2_PO_4_ (50 mM), pH 7.4, 25 °C ± 2 deg. C. The post-mixing DHE concentration displaces 97.5% of CH or CS from Aβ_42_; hence, the displacement reactions are pseudo-first order. Progress curves were determined in triplicate and k_off_ was obtained by least-squares fitting the averaged curves to a single-exponential equation using Pro-K.

#### Aβ-fibril formation

Fibril formation was monitored *via* the binding-induced change in ThT fluorescence (λ_ex_ = 450 nm, λ_em_ = 482 nm) (76). Reaction conditions: Aβ_42_ (1.0 μM), ThT (30 μM), ligand (CH (3.0 μM, 17 × K_d_) *or* CS (1.6 μM, 17 × K_d_) *or* no ligand), DMSO (0.50 % v/v), K_2_PO_4_ (50 mM), pH 7.4, 25 °C ± 2 deg. C. Reagents (peptide, CH, CS, and ThT) were prepared immediately prior to use. CH and CS were solubilized in neat DMSO. Progress curves were determined in triplicate, and data were fit using the Nucleation-Fragmentation-Elongation (NEF) model (see Results and discussion).

#### Aβ_42_ nucleation molecularity

The formation of fibril nuclei was monitored *via* the Aβ-binding induced change in ThT fluorescence (λ_ex_ = 450 nm, λ_em_ = 482 nm, (42)). Reactions were initiated by mixing peptide and ligand (CH or CS) under conditions where the free-ligand concentration was maintained at 17 × K_d_ by setting the [ligand] = [peptide] + 17 × K_d_. The post-mixing peptide and ligand concentrations were as follows: *unliganded Aβ*_*42*_ - [Aβ_42_] = 1.0, 2.0, 3.0 or 4.0 μM; *CH•Aβ*_*42*_ - [Aβ_42_] = 0.5, 1.0, 1.5 or 2.0 μM, [CH] = 2.5, 3.0, 3.5 or 4.0 μM; *CS•Aβ*_*42*_ - [Aβ_42_] = 0.25, 0.5, 0.75 or 1.0 μM, [CS] = 0.85, 1.1, 1.35 or 1.60 μM. The reaction solution was equilibrated at 25 °C ± 2 deg. C and contained ThT (30 μM), DMSO (0.5% v/v), and K_2_PO_4_ (50 mM), pH 7.4. CH, CS, and ThT were dissolved in neat DMSO immediately prior to use. The nucleation rate for each condition was obtained by a linear least-squares fitting of the very early region of the fibril formation lag phase. All fits were initiated at t_0_ and deviation from linearity was within experimental error (*i.e.*, χ^2^ > 0.95). Measurements were performed in triplicate and plotted *versus* [Ligand•Aβ_42_]^2^. Rate constants were obtained by linear least-squares fitting of the Rate-*versus*-[L•Aβ_42_]^2^ plots (54) and agree well with the nucleation rate constant obtained from NEF-fits of the full progress curve (see Table 2).

### Molecular dynamics simulations

#### Solvent equilibration of unliganded Aβ_42_, CH, and CS

Initial atomic coordinates for the Aβ_42_ model were obtained from the Aβ_42_ NMR structure (1IYT, (57)); missing atoms were added using SWISS MODELLER (72). The Aβ_42_ model, CH, and CS were protonated (pH 7.4) and energy-parameter files (pH 7.4) were created using ATB (73, 77). Molecules were parameterized with GROMASS57 (78, 79) and energy was minimized using GROMACS (55, 56). Molecules were centered in a 5.0 × 5.0 × 5.0 nm cube of water containing PO_4_ (50 mM) and KCl (0.10 M) at pH 7.4, 298 K, and thermally equilibrated in 100 ps increments. Simulations are considered to reach equilibrium when the time-averaged RMSD over 0.5 ns intervals no longer change with time (80). All analyses were performed with equilibrated models. Interaction-energy and cluster analyses were performed using GROMACS *g_energy* and *g_cluster* functions. Structures were visualized using PyMol (81). Expanding simulation cube sizes from 5.0 × 5.0 × 5.0 nm to 7.0 × 7.0 × 7.0 nm did not affect outcomes.

#### Ligand docking

Aβ_42_ and ligands were semi-randomly (82) positioned in a 5.0 × 5.0 × 5.0 nm cube of water, PO_4_ (50 mM), and KCl (0.10 M) at pH 7.4, 298 K with the constraint that the smallest inter-atom distance between the two structures was ≥5 Å. The ligand was thermally equilibrated in 100 ps increments, and the energy-of-interaction was calculated over 20 ns using *g_energy*. Binding free energies were calculated by subtracting the energy-of-interaction of the ligand-bound Aꞵ_42_ from the sum of the individual ligand and peptide interaction energies (32, 33).

#### Aβ_42_ dimerization

Pairs of monomeric Aβ_42_-ligand models generated *via* dynamic docking were semi-randomly placed in a 10 × 10 × 10 nm cube of water, PO_4_ (50 mM), and KCl (0.10 M) at pH 7.4, 298 K, with the constraint that the smallest inter-atom distance between the two structures was ≥10 Å. Each Aβ_42_•CH cluster was maintained within its cluster by a weak restraining force (0.50 kJ/mole) that switched to 0 kJ/mole once the monomer/monomer interaction energy rose to one 10th the energy needed to separate monomers in an oligomer (25 kJ per mole) (64). The systems were thermally equilibrated in 100 ps increments and simulations were run for 5.0 μs.

#### Aβ_42_ oligomerization

Eight Aβ_42_•CS monomers, obtained from docking studies, were semi-randomly positioned in a 10 × 10 × 10 nm cube containing water, PO_4_ (50 mM), and KCl (0.10 M), pH 7.4, 298 K, with the constraint that the smallest inter-atom distance between any two structures was ≥10 Å. The system was thermally equilibrated in 100-ps increments and the simulation was run for 50 ns.

#### Aβ_42_ fragmentation

A 25-mer was constructed by “stitching together” and energy-minimizing 8-mers obtained from the oligomerization studies. A 25-mer Aβ_42_•CS fibril was placed in a 20 × 20 × 20 nm cube of water, PO_4_ (50 mM), and KCl (0.10 M), pH 7.4, 298 K. The system was thermally equilibrated in 100 ps increments and the simulation was run for 500 ns. Fragmentation was monitored by increased solvent exposure at the subunit interfaces.

## Data availability

All data and materials are available upon request (email: tom.leyh@einsteinmed.org).

## Supporting information

This article contains supporting information.

## Conflict of interest

The authors declare no competing financial interest.
