# Supplementary material for: Sterol-activated amyloid beta fibril formation
Source: J Biol Chem. 2023 Nov 8;299(12):105445. doi: 10.1016/j.jbc.2023.105445 (PMC10704437; doi:10.1016/j.jbc.2023.105445)
Supplement: Supplemental Figs. S1–S4 [file mmc1.docx]

**Figure S1**

**Figure S1*.*** *Fibril Formation Controls for Equilibrium-Binding Studies.* **Panel A.** *Figure 1A Control.* Fibril formation was initiated by addition of freshly prepared Aβ_42_ (200 nM) to a solution containing DHE (10 nM), ThT (30 µM), K_2_PO_4_ (50 mM), pH 7.4, 25 ± 2 C°. **Panel B.** *Figure 1B Control.* Fibril formation was initiated by addition of freshly prepared Aβ_42_ (3.0 µM) to a solution containing DHE (1.0 µM), ThT (30 µM), K_2_PO_4_ (50 mM), pH 7.4, 25 ± 2 C°. **Panels C and D.** *Figure 1C and 1D Controls.* Fibril formation was initiated by addition of freshly prepared Aβ_42_ (20 nM) to a solutions containing CH (5.0 µM, Panel C) or CS (2.0 µM, Panel D), DHE (100 nM), ThT (30 µM), K_2_PO_4_ (50 mM), pH 7.4, 25 ± 2 C°. Fibril formation was detected *via* the fluorescence change associated with ThT binding (λ_ex_ = 450 nm, λ_em_ = 482 nm) and is reported as the percentage of ThT signal at reaction endpoint. Each progress curve was performed in triplicate and the averaged data are shown.

**Figure S2**. *The Six Stable Forms of CH•Aβ_42_*. Structures are numbered as described in *Results and Discussion*. Beneath each form is the percent of the time the form was present during 200 ns MD simulations (see, *Results and Discussion*). All residues in direct contact with ligand are shown in “stick” and labeled. Small red spheres mark the Aβ_42_ peptide C‑terminal residue, A42.

**Figure S2**

**Figure S3**. *Homo- and Heterodimer Formation using Dimerization Competent Monomers*. Dimer formation was simulated using CS•Aβ_42_ and Form 3 CH•Aβ_42_ monomers. Simulations were performed as described in *Methods*, *Aβ_42_ Dimerization Simulations*. The red (CH) and black (CS) dimerization data sets are also shown in Figure 7B (main text). The dimerization rates are indistinguishable.

**Figure S3**

**Figure S4**

**Figure S4**. *Fragmentation of the* *CS•Aβ_42_* *Fibril*. A 25-mer CS•Aβ_42_ fibril was constructed by extending the 8-mer generated in the oligomer formation studies (see, *Results and Discussion*). The 25-mer was energy minimized using *steepest_decent* and positioned in the center of 10 x 10 x 10 nm cube containing water, PO_4_ (50 mM) and KCl (0.10 M), pH 7.4, 25 °C. Approximately 10 ns after initiating the simulation, the 25-mer spontaneously and nearly simultaneously fragmented into three segments (a 10-mer (green), 8‑mer (red), and 7-mer (blue)).
